# Diagnosis and management of primary ciliary dyskinesia

**DOI:** 10.1186/s13630-014-0011-8

**Published:** 2015-01-22

**Authors:** Claudius Werner, Jörg Große Onnebrink, Heymut Omran

**Affiliations:** Department of General Pediatrics, Pediatric Pulmonology Unit, University Children’s Hospital Muenster, Albert-Schweitzer-Campus 1, Geb. A1, D-48149 Münster, Germany

## Abstract

Primary ciliary dyskinesia (PCD) is a rare autosomal recessive disorder with defective structure and/or function of motile cilia/flagella, causing chronic upper and lower respiratory tract infections, fertility problems, and disorders of organ laterality. Diagnosing PCD requires a combined approach utilizing characteristic phenotypes and complementary methods for detection of defects of ciliary function and ultrastructure, measurement of nasal nitric oxide and genetic testing. Currently, biallelic mutations in 31 different genes have been linked to PCD allowing a genetic diagnosis in approximately ~ 60% of cases. Management includes surveillance of pulmonary function, imaging, and microbiology of upper and lower airways in addition to daily airway clearance and prompt antibiotic treatment of infections. Early referral to specialized centers that use a multidisciplinary approach is likely to improve outcomes. Currently, evidence-based knowledge on PCD care is missing let alone management guidelines. Research and clinical investigators, supported by European and North American patient support groups, have joined forces under the name of BESTCILIA, a European Commission funded consortium dedicated to improve PCD care and knowledge. Core programs of this network include the establishment of an international PCD registry, the generation of disease specific PCD quality of life questionnaires, and the first randomized controlled trial in PCD.

## Review

The term primary ciliary dyskinesia (PCD (MIM 244400)) has been used for a clinically and genetically heterogeneous group of recessive disorders with defective ciliary motility resulting in chronic upper and lower respiratory tract disease. Organ laterality defects occur in approximately ~50% of cases, usually situs inversus totalis (Kartagener’s syndrome). In 1976, Afzelius reported that PCD is characterized by ultrastructural defects of cilia leading to ‘immotile’ cilia [1]. Subsequent research lead to the replacement of the term ‘immotile cilia syndrome’ by ‘primary ciliary dyskinesia’ in order to emphasize that not only ciliary immotility but also abnormal ciliary motility causes PCD [2]. Even this term seems to be inappropriate to cover all disease variants, as demonstrated by the recent identification of a disease entity characterized by typical, severe clinical PCD features (without situs abnormalities) caused by defective generation of multiple motile cilia [3,4]. This review provides a state-of-the art overview on diagnosis and management of PCD. It augments knowledge summarized in recent reviews [2,5-10]. Due to the thematic overlap, partial similarities to these reviews are inevitable.

### Epidemiology

The PCD prevalence is difficult to determine [11] and is in the range of 1:4,000 to <1:50,000 [12]. Most likely, this rather reflects differences in access to diagnostic facilities as several complex diagnostic tests, which are not universally available, have to be combined for diagnosing PCD. On the other hand, some degree of variability can be explained by a higher prevalence in societies with a high degree of consanguinity [13].

#### Diagnosis

**Figure 1:**
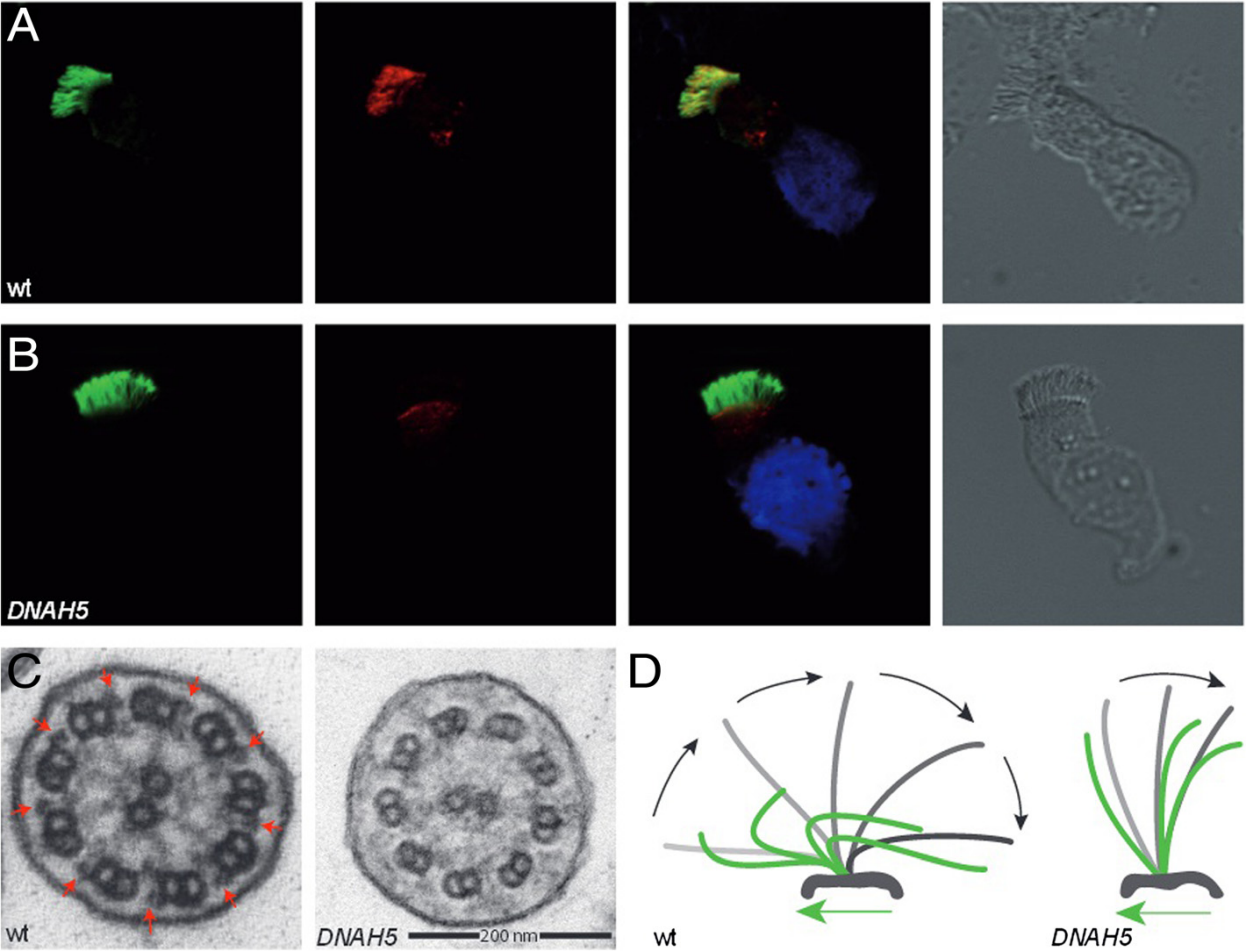
**Methods used for PCD diagnosis.** **(A, B)** Immunofluorescence co-staining of human respiratory epithelial cells with DNAH5-specific antibodies (red) and antibodies against acetylated α-tubulin (green). Nuclei were stained with Hoechst 33342 (blue). Overlays and bright-field images are shown on the right. Whereas in healthy human respiratory epithelial cells (wt, A) both DNAH5 and acetylated α-tubulin antibodies co-localize along the entire length of the ciliary axonemes, in an individual with an outer dynein arm defect **(B)**, the ODA heavy chain DNAH5 is absent from the axonemes. **(C)** Transmission electron tomography of healthy respiratory epithelial cells (wt) showing no ultrastructural abnormality. Outer dynein arms (ODAs) are highlighted with red arrows. In an individual with *DNAH5* mutations, ODAs are missing. **(D)** Diagram of ciliary beat patterns as deduced from high-speed videomicroscopy. A normal ciliary beat pattern (wt) is characterized by a strong beating stroke (symbolized in grey) followed by a recovery stroke (symbolized in green). In *DNAH5* mutant cilia, only a minimal residual ciliary activity is present.

PCD is both under-diagnosed and diagnosed too late [12]. Therefore, clinicians should increase their level of suspicion for PCD in patients with typical phenotypes (Table 1). Diagnosing PCD requires a combined approach of complementary methods (Table 2; Figure 1), all of which have limitations [2,14]. As most institutions do not have adequate resources for a thorough diagnostic evaluation, referral to specialized centers is strongly recommended [2,6,11].

**Table 1:** **Candidates for primary ciliary dyskinesia (PCD) testing (adapted from** [5,11]**)**

1.	Individuals with situs inversus totalis or other situs abnormalities	
2.	Individuals with both upper and lower respiratory tract disease	
	a.	Upper airways disease includes: chronic rhinitis/nasal discharge, chronic sinusitis, hearing impairment due to glue ear, chronic otitis media
	b.	Lower airways disease includes: chronic wet cough, atelectasis or bronchiectasis, notably in middle lobe, lingula or lower lobes, chronic/recurrent bronchitis/pneumonia
3.	History of unexplained neonatal respiratory distress	
4.	Positive family history (for example, affected sibling)	
5.	Congenital heart defect, notably if upper/lower airways disease and heterotaxy are present

**Table 2:** **Methods and limitations used for confirmation of PCD diagnosis**

**Method**	**Limitation**
Nasal NO level	May be decreased in other disorders, for example, acute sinusitis or cystic fibrosis; rarely normal values may be present in PCD
High frequency video microscopy (HVMA)	Variants with subtle beating abnormality may be interpreted as normal; secondary ciliary dyskinesia due to infection and inflammation is very common - distinction from PCD phenotype may be difficult
Transmission electron microscopy (TEM)	Approximately ~30% of PCD cases have no ultrastructural abnormality; false-positive diagnoses common in some variants (notably inner dynein arm defects)
Immunofluorescence microscopy (IF)	No abnormality in approximately ~20%; technical difficulties if specimen contains a lot of mucus
Genetics	Expensive due to high number of PCD genes; only approximately 60% of cases can be identified by genetic testing at present

### Clinical phenotype

**Figure 2:**
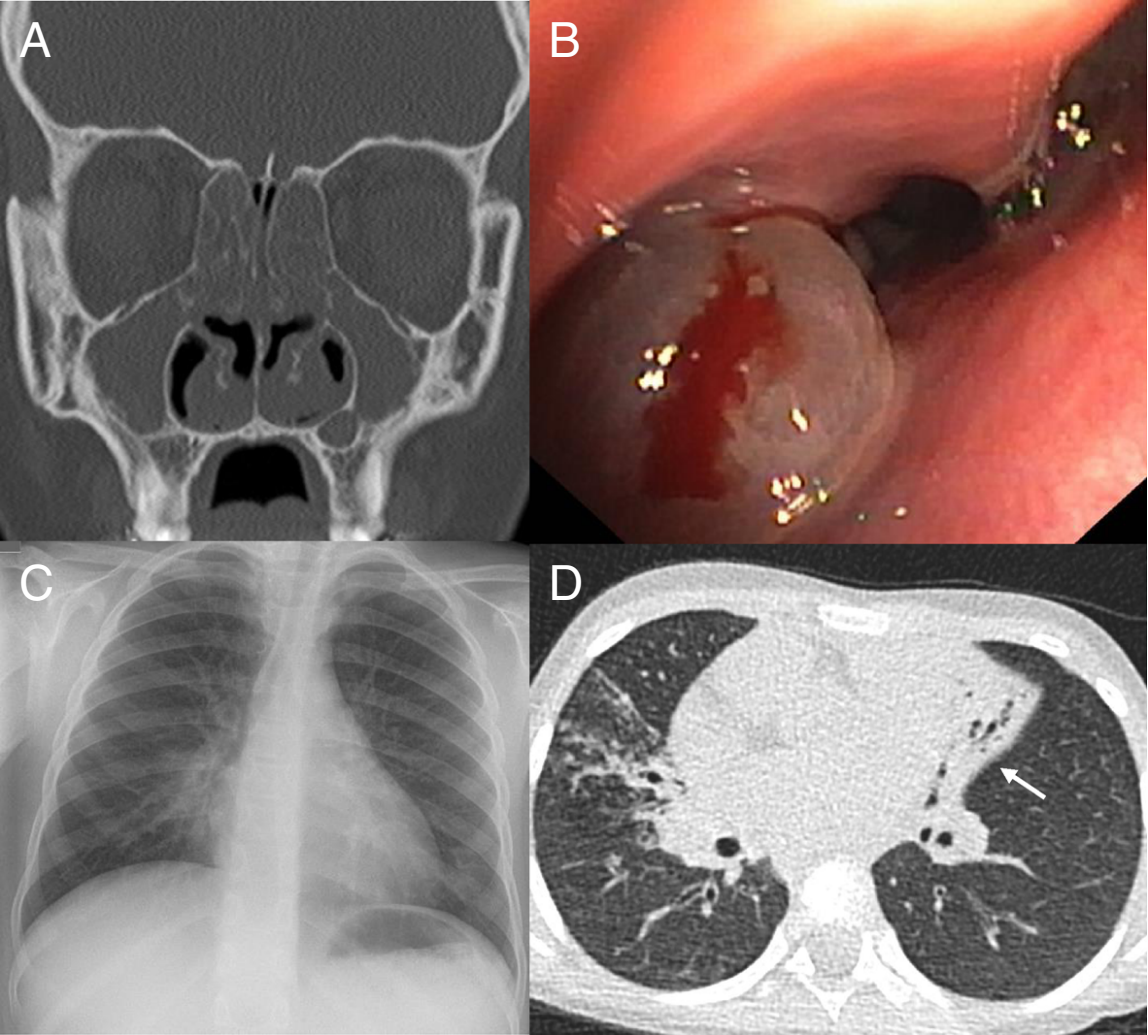
**Clinical features of primary ciliary dyskinesia. (A)** Coronal computed tomography (CT) scan of a 17-year-old PCD individual showing diffuse pansinusitis with mucosal thickening and polyposis. **(B)** Endoscopic view showing nasal polyp (same patient). **(C)** Chest X-ray of a 6-year-old PCD individual with middle lobe atelectasis. Silhouetting of the right heart border is present. **(D)** Chest CT of a 6-year-old individual with situs inversus totalis. The left-sided middle lobe shows extensive bronchiectasis with volume loss (white arrow). In addition, consolidations and mucous impaction are present in the right upper lobe.

Up to 85% of individuals with PCD have a history of unexplained neonatal respiratory distress [15]. Symptoms comprise mild transient tachypnoea, atelectasis, and can advance to respiratory failure requiring ventilatory support. Directly after birth, neonates with PCD present with persistent rhinitis or a blocked nose leading to feeding difficulties. Chronic rhinosinusitis develops in childhood and lasts through adulthood (Figure 2). Already during infancy, conductive hearing loss frequently occurs due to middle ear effusion that may progress to glue ear. Infants typically develop daily wet cough and recurrent upper and lower airways infections. Although a life-long symptom, patients often do not report cough as under-recognition due to adaptation to this ever-present symptom or cough suppression due to embarrassment are common. Consolidation, atelectasis, and bronchiectasis are constant findings in adults but may be present already in infancy (Figure 2) [15]. Typically, the middle and lingula lobes of the lung are affected predominantly followed by the lower lobes. Involvement of the upper lobes usually occurs at a later disease stage [16].

In many PCD variants, ciliary dysfunction also involves cilia of the embryonic node which are essential for establishing the left-right asymmetry of visceral organs. Dysfunction of these nodal cilia results in a randomization of left-right body asymmetry. Hence, 40% to 50% of PCD individuals present with situs inversus totalis, a condition known as Kartagener’s syndrome, and a smaller subset of individuals (at least 6.3%) display complex situs anomalies associated with congenital heart disease [17]. Of note, respiratory symptoms are very common in patients with heterotaxy and complex cardiac disease due to the heart defect [18]. Thus, these patients have a high risk that PCD, a chronic respiratory disease requiring specific management, may be overlooked.

As the structure of sperm flagella is similar to the ciliary axoneme, many PCD variants are associated with male infertility. By contrast, it is yet unclear to what extent women with PCD are affected by subfertility due to dysfunction of cilia in the fallopian tubes.

#### Nasal nitric oxide

Measuring nasal nitric oxide (nNO) is a very robust screening test for PCD as values are very low in most PCD patients [19-21]. nNO is analyzed by placing a nasal olive probe into one nostril with a tube connecting the olive with the analyzer. The measurement is done while the patient performs an exhalation against resistance manoeuver in order to close the soft palate and thereby separate the sinonasal compartment from the bronchial airflow. Among several commercially available NO analyzers, most data on reliability and validity exist with chemiluminiscence analysers. Using such a device, a nNO production rate less than 77 nL/min has a sensitivity and specificity of 0.98 and >0.999, respectively, for PCD diagnosis [20]. As breathing manoeuvers are difficult particularly for young children, simpler tests such as tidal breathing sampling are currently evaluated [22,23]. nNO should not be used as single diagnostic test because low levels can also be present in cystic fibrosis (CF), sinusitis, nasal polyposis, and acute upper airways infections and because normal nNO levels rarely have been reported in PCD patients [21].

#### High-speed video microscopy

High-speed video microscopy (HVMA) of ciliary beat pattern and frequency of respiratory epithelial cells obtained by trans-nasal brushing currently is the first-line diagnostic test in many centers. Cilia can be observed at high-resolution in real time and with slow-motion replay [24]. HVMA is performed directly after obtaining the specimen and immediately yields a definite diagnosis in a subset of cases. Ciliary beat abnormalities include static cilia, almost static cilia with minimal movements, stiff beating due to a reduced bending capacity/amplitude, abnormal circular beating and hyperkinetic cilia. These patterns are linked with specific ultrastructural defects and genetic variants [24]. Thus, HVMA findings can guide subsequent analyses revealing the underlying molecular defect. HVMA though is a challenging method for various reasons: (1) HVMA protocols differ among centers in many aspects: sampling techniques, microscopes and cameras, temperature during analysis, software, and evaluation criteria [24]; (2) Whereas ciliary beat frequencies can be determined easily, development of objective methods to reliably distinguish PCD-specific ciliary beat pattern abnormalities from normal findings or secondary damage are at a very provisional stage [25]. Thus, HVMA evaluation remains strongly dependent on the experience of the investigator; (3) Recent molecular findings have revealed PCD variants that may easily be misinterpreted as normal [26,27] or difficult to assess due to lack of ciliated tissue caused by defects of multiple motile cilia generation [3,4]; (4) Acute or chronic infection and inflammation - very common both in PCD and non-PCD individuals - lead to secondary ciliary dyskinesia that may be difficult to distinguish from primary ciliary dyskinesia [2,28]. To overcome diagnostic difficulties related to secondary abnormalities caused by infection and inflammation, HVMA as well as transmission electron microscopy (TEM; see below) or immunofluorescence analysis (IF; see below) can be performed additionally after *in vitro* ciliogenesis in respiratory epithelial cell cultures. Although the ciliary beating phenotype may be altered after cell culture, primary abnormalities are still present [29,30].

#### Assessment of ciliary composition by transmission electron microscopy or immunofluorescence analysis

Analysis of ciliary cross-sections by TEM has been used traditionally to confirm a PCD diagnosis. However, as this approach cannot be used to identify an increasing number of PCD variants (at least 30%; [31,32]) with (near) normal ultrastructure, this approach can no longer be the ‘gold standard’ for diagnosis. Defects that can reliably be identified by TEM include a complete or partial absence of outer dynein arms (ODAs; Figure 1), combined ODA and inner dynein arm (IDA) defects, and microtubular disorganization defects. Isolated IDA defects should not be diagnosed by a single TEM analysis alone as false-positive diagnoses commonly occur with isolated IDA defects [33]. Only a subset of radial spoke defects can be diagnosed by TEM analyses [34]. Defects of nexin link components [26,35], central pair components [27], ciliary biogenesis defects [3,4] and defects caused by *DNAH11* mutations [31,36] usually cannot be identified by routine TEM analysis.

During the last decade, high resolution IF has been introduced as an additional tool to investigate the subcellular localization of the ciliary proteins in human respiratory epithelia [37]. Meanwhile, it is possible to reliably identify all ultrastructural abnormalities detectable by TEM, such as outer dynein arm defects (Figure 1) or microtubular disorganization with IDA defect [37-39], and additionally abnormalities of nexin links components [26] and radial spoke head proteins [40,41]. The technology has been adopted by several laboratories and it is likely that further development will allow recognizing an increasing number of PCD variants.

#### Genetics

PCD is an autosomal recessive disorder. Mutations in a rapidly expanding number (currently 31) of genes are disease-causing (Table 3). The majority of mutations are loss-of-function variants, while missense mutations can be found in a minority of cases. Most mutations are private. Clustering of mutations in specific genetic regions is less common than in other genetic disorders. The specific gene mutations correlate to their structural and video microscopic phenotype.

**Table 3:** **Genes associated with PCD and corresponding ultrastructure**

**Gene**	**Reference**	**Axonemal/cellular structure or function**	**Routine TEM**		**Routine IF**	
			**Informative** ^**a**^	**Finding**	**Informative** ^**a**^	**Abnormal staining with antibodies against**
*DNAH5*, *DNAI1*, *DNAI2*, *DNAL1*, *NME8 (TXNDC3)*	[42-46]	ODA subunit	✓	ODA-defect	✓	ODA component
*CCDC114*, *ARMC4*, *CCDC151*	[47-49]	ODA targeting/docking factor	✓	ODA-defect	✓	ODA component
*DNAAF1 (LRRC50)*, *DNAAF2 (KTU)*, *DNAAF3*, *HEATR2*, *LRRC6*, *ZMYND10*, *DYX1C1 (DNAAF4)*, *SPAG1*, *CCDC103*, *C21ORF59*	[38,50-58]	Cytoplasmic dynein arm assembly or transport factor	✓	IDA + ODA defect	✓	ODA component + IDA component
*RSPH1*, *RSPH4A*, *RSPH9*	[40,59]	RSPH subunit	**(**✘**)**	Missing CP or TTD; often normal	✓	RSPH components
*CCDC39*, *CCDC40*	[39,60]	NL/DRC factor	✓	microtubular disorganisation + IDA-defect	✓	DRC components + IDA components
*CCDC164*, *CCDC65*	[26,58]	NL subunit	✘	NL defect only rarely discernible	✓	NL components
*DNAH11*	[36]	ODA subunit	✘	Normal	✘	
*HYDIN*	[27]	CP subunit	✘	Normal (C2b absence only visible in TEM tomography)	✘	
*CCNO*, *MCIDAS*	[3,4]	*CCNO*: cytoplasmic centriole assembly and docking factor; MCIDAS: nuclear regulator of *CCNO* and *FOXJ1*	**(**✘**)**	Usually misinterpreted as secondary ciliary aplasia; reduced numbers of MMC; basal bodies and rootlets are mislocalized	**(**✘**)**	Usually misinterpreted as secondary ciliary aplasia; *MCIDAS:* lack of any axonemal components *CCNO*: Rootletin mislocalization, CCNO deficiency
*OFD1*, *RPGR*	[61,62]	Functions related to non-motile cilia; role in motile cilia unknown	✘	Normal/unspecific	✘	

^a^Informative denotes: detectable in routine diagnostics.

CP, central pair tubuli; DRC, dynein regulatory complex; IDA, dynein arm; IF, immunofluorescence microscopy; MMC, multiple motile cilia; NL, nexin link; ODA, outer dynein arm; RSPH, radial spoke head; TEM, transmission electron microscopy; TTD, tubular transposition defect (8 + 1 structure).

Only preliminary evidence correlates genetic findings with distinct clinical phenotypes. Mutations affecting the composure of the central pair (*HYDIN* [27]) or radial spokes (*RSPH1* [34,40], *RSPH4A*, *RSPH9* [59]) as well as the generation of multiple motile cilia (*MCIDAS* [3], *CCNO* [4]) do not result in situs abnormalities. Patients with mutations in *RSPH1* may have a milder clinical course [34]. Males with mutations in *CCDC114* are not affected by infertility due to sperm immotility [47]. Subjects with reduced generation of multiple motile cilia may have a more severe respiratory disease with lung failure at younger age [3,4].

Modern high-throughput genetic technologies allow identification of disease-causing biallelic mutations in approximately ~60% of patients. Although not yet implemented for routine diagnostics, next generation sequencing already is cost-efficient and effective in diagnosing PCD compared to traditional sequential Sanger sequencing of single genes. However, it has to be kept in mind that every year novel genetic defects are discovered and therefore genetic testing cannot be used to rule out a PCD diagnosis. In addition, expertise is necessary to distinguish mutations from rare polymorphisms.

#### Establishing a PCD diagnosis

Given the heterogeneity of possible findings associated with PCD, there is no uniform approach in diagnosing PCD. Currently, we consider a PCD diagnosis confirmed if the following diagnostic criteria are fulfilled: (1) clinical presentation consistent with PCD; and (2) confirmation of the diagnosis by at least two of the following methods: unequivocally abnormal HVMA finding, unequivocally abnormal TEM finding, unequivocally abnormal IF finding, abnormally low nNO concentration/production and demonstration of unequivocal biallelic disease-causing mutations by genotyping. In cases where only HVMA and nNO concentration/production are abnormal, HVMA should be repeated at least three times and show the same abnormal results each time. Individuals with typical clinical symptoms and only one abnormal diagnostic test are usually considered to have a possible PCD diagnosis with exceptions made on an individual basis (for example, identification of the same biallelic disease-causing mutations in a subject of a sibling with a confirmed diagnosis).

Of note, this approach is provisional, as it is very likely that further research will continue to modify our understanding of different PCD phenotypes.

### Management

There is a lack of evidence-based management guidelines for PCD. Randomized controlled trials have not yet been performed in this condition. Thus, therapies are deduced from other diseases with defective mucociliary clearance, notably CF and non-CF bronchiectasis. The PCD task force of the European Respiratory Society has issued recommendations for the management of PCD highlighting the importance of routine airway clearance techniques, the use of antibiotics to control infection, and the avoidance of harmful agents such as active and passive smoke [11].

To overcome limitations in PCD care, European and North American investigators and clinicians, as well as patient support groups, have joined forces in BESTCILIA, a European Commission funded consortium dedicated to improve PCD care and knowledge (http://www.bestcilia.eu/). Core programs of this network include: (1) A cross-sectional observational trial for answering pertinent questions on clinical phenotype, severity, prognosis, and effect of treatments on outcomes; (2) An international prospective PCD registry for systematic data collection on incidence, clinical presentation, treatments, and course of the disease. This will allow to monitor trends in management and outcomes and to recruit patients for trials. (3) The introduction of standardized diagnostic testing for PCD in three European countries (Greece, Poland, Cyprus), where this is currently not available. This approach will be paradigmatic for other countries how to implement sophisticated diagnostic facilities; (4) The development of PCD-specific health-related quality of life questionnaires (QoL-PCD) as an outcome measure in clinical trials. QoL-PCD is furthermore particularly useful to track changes over time and therefore to assess changes in disease impact on daily life. Therefore, QoL-PCD will be integrated into the longitudinal international PCD registry; (5) The first randomized controlled clinical trial ever performed in PCD to analyze the efficacy and safety of long-term use of azithromycin.

#### Monitoring

Ideally, individuals with PCD should be followed up in specialized centers every 3 months for spirometry, microbiological studies of both upper and lower airways secretions, and review of respiratory therapy techniques.

Spirometry is easy to perform, but it is an insensitive marker of lung function decline, particularly in younger patients. Therefore, assessment of disease severity using high-resolution computed tomography (HRCT) should be considered at larger intervals [63]. Magnetic resonance imaging (MRI) protocols have been developed showing good agreement with HRCT for determining extent and severity of lung disease in non-CF bronchiectasis [64]. Although inferior to HRCT with regard to speed, image contrast and spatial resolution, MRI is an excellent radiation-free tool especially for longitudinal analyses.

Possibly, determination of lung clearance index (LCI) using multiple breath washout may evolve as a tool to monitor PCD lung disease accurately and non-invasively. It correlates well with HRCT findings in CF and is more sensitive to early changes in lung physiology. However, a recent study has failed to demonstrate a correlation between LCI and HRCT scores in PCD [65].

Monitoring of upper airways disease includes regular hearing tests that should be performed at least every 6 months in young children and every year in adults. PCD individuals are at an increased risk to develop sleep disordered breathing, particularly due to obstructive sleep apnea syndrome [66,67]. Even if there are currently no evidence-based data on choice and efficacy of possible treatment modalities, assessment of sleep disorders should be part of a PCD management plan.

#### Lower airways management

Daily airway clearance and aggressive antibiotic treatment of respiratory tract infections are treatment cornerstones of PCD lung disease. Review of individualized, age-appropriate airway clearance therapies should be part of the regular follow-up visits. No individual technique has proven superior. Although of unproven benefit, inhalation of hypertonic saline to improve cough clearance is widely used as is use of bronchodilators. Physical exercise effectuates general health improvements and therefore is encouraged. The role of inhaled rhDNase, a medication commonly used in CF, is unclear. In non CF-bronchiectasis, rhDNase has been shown ineffective and therefore it is not regularly used in PCD [68]. Consistent with this approach, PCD patients usually do not report changes in sputum viscosity after rhDNase inhalation. Individuals with PCD and clear evidence of coexisting asthma can be treated with inhaled corticosteroids. Otherwise, inhaled corticosteroids should be avoided as they might bear the risk of an increased susceptibility to infections.

Antibiotic treatment of respiratory tract infections should be instituted promptly and adjusted to microbiological findings. Fever is not a reliable symptom in many PCD individuals. We recommend antibiotics if patients note an increase in sputum quantity or a change in sputum viscosity or color. The most commonly cultured pathogens are *Haemophilus influenzae*, *Staphylococcus aureus*, *Moraxella catarrhalis*, and *Streptococcus pneumoniae* [69]. Chronic *Pseudomonas aeruginosa* is found particularly in adults with advanced disease. It is unclear, if chronic *Pseudomonas aeruginosa* infection has similar detrimental effects on lung function in PCD as in CF. However, as prompt eradication of potentially harmful bacteria remains a core goal in PCD management, protocols based on those used in CF are recommended. Chronic *Pseudomonas aeruginosa* infection is often treated with nebulized antibiotics. Some centers advocate the use of long-term prophylactic antibiotics. Currently, there is no evidence for this approach. Empirical knowledge, however, supports long-term antibiotic use in children requiring frequent courses of antibiotics. Currently, a BESTCILIA trial is recruiting patients to test the effects of long-term Azithromycin use.

Lung surgery in PCD is usually not recommended; in selected cases of localized disease refractory to conservative management, lobectomy can be considered. Bilateral lung transplantation is a treatment option for end-stage lung failure.

#### Upper airways management

Impaired mucociliary clearance in the upper airways affects the nasal cavity, the paranasal sinuses, and the middle ear. Persistent nasal discharge and blockage is treated by nasal irrigation. Chronic rhinosinusitis may be treated additionally by sinonasal inhalation of hypertonic saline solution and, when infective exacerbations occur, with antibiotics. Sinus surgery for nasal polyposis bears a high risk of early recurrence and is therefore performed only in cases of severely blocked passages. Similar to the treatment of lower airways disease, topical corticosteroids are useful only in individuals with additional allergic rhinosinusitis. The role of ventilation tubes for treatment of conductive hearing loss due to chronic otitis media with effusion is controversial. Due to the impaired mucociliary clearance, PCD patients have a higher risk of developing chronic mucopurulent discharge after ventilation tube insertion [11,70]. Therefore, hearing aids are preferred to manage hearing loss. By contrast, a recent report suggests that hearing may be improved by ventilation tubes, and otorrhea can be controlled [71]. These controversial reports highlight the lack of evidence based medical approaches.

#### Non-respiratory manifestations

Congenital heart disease, when present, usually requires corrective or palliative surgery. Male or female infertility is managed with adequate reproductive techniques. However, as men with PCD are not always infertile and women only have a slightly decreased fertility (if at all), birth control measures are necessary if there is no wish to have children.

## Conclusions

Many PCD individuals receive suboptimal management because there are no evidence-based treatment guidelines. Establishing the diagnosis requires several complementary tests. A multidisciplinary management approach is well recognized to benefit long-term outcomes. Regular surveillance should include lung function testing, microbiological studies, and review of airway clearing techniques. Currently, management is mainly deduced from evidence from other suppurative lung diseases.

## Abbreviations

CP: Central pair tubuli
CF: Cystic fibrosis
DRC: Dynein regulatory complex
HRCT: High-resolution computed tomography
HVMA: HIGH-speed Video microscopy
IDA: Inner dynein arm
IF: Immunofluorescence
LCI: Lung clearance index
MMC: Multiple motile cilia
MRI: Magnetic resonance imaging
NL: Nexin link
nNO: Nasal nitric oxide
ODA: Outer dynein arm
PCD: Primary ciliary dyskinesia
RSPH: Radial spoke head
TEM: Transmission electron microscopy
TTD: Tubular transposition defect
